# Biased Saccadic Responses to Emotional Stimuli in Anxiety: An Antisaccade Study

**DOI:** 10.1371/journal.pone.0086474

**Published:** 2014-02-11

**Authors:** Nigel T. M. Chen, Patrick J. F. Clarke, Tamara L. Watson, Colin MacLeod, Adam J. Guastella

**Affiliations:** 1 Brain and Mind Research Institute, University of Sydney, Camperdown NSW, Australia; 2 School of Psychology, University of Western Australia, Crawley WA, Australia; 3 School of Social Sciences and Psychology, University of Western Sydney, Penrith NSW, Australia; Bellvitge Biomedical Research Institute-IDIBELL, Spain

## Abstract

Research suggests that anxiety is maintained by an attentional bias to threat, and a growing base of evidence suggests that anxiety may additionally be associated with the deficient attentional processing of positive stimuli. The present study sought to examine whether such anxiety-linked attentional biases were associated with either stimulus driven or attentional control mechanisms of attentional selectivity. High and low trait anxious participants completed an emotional variant of an antisaccade task, in which they were required to prosaccade towards, or antisaccade away from a positive, neutral or threat stimulus, while eye movements were recorded. While low anxious participants were found to be slower to saccade in response to positive stimuli, irrespectively of whether a pro- or antisaccade was required, such a bias was absent in high anxious individuals. Analysis of erroneous antisaccades further revealed at trend level, that anxiety was associated with reduced peak velocity in response to threat. The findings suggest that anxiety is associated with the aberrant processing of positive stimuli, and greater compensatory efforts in the inhibition of threat. The findings further highlight the relevance of considering saccade peak velocity in the assessment of anxiety-linked attentional processing.

## Introduction

Information processing approaches to the study of cognition and emotion suggest that high trait anxiety is maintained by biases in early attentional processing [1], [2]. A large base of research has demonstrated a consistent association between anxiety and attentional bias towards the processing of threatening information [3]. In addition, studies which have experimentally manipulated such a bias have further shown a causal link between attentional processing and anxiety vulnerability [4]–[6].

In addition to biased attention to threat, a growing base of literature suggests that anxiety is also associated with the deficient attentional processing of positive information [7], [8]. While individuals low in trait anxiety may preferentially process positive information [9], attentional assessments such as the dot probe task, have shown an association between anxiety and a reduced attentional preference to positive stimuli [10]. Anxiety has additionally been associated with the attentional neglect of positive cues during a speech task stressor [11], [12]. Recent eye movement research has further demonstrated that clinically anxious individual are faster to disengage their attention from positive stimuli [13]. Moreover, deficient attentional processing of positive stimuli may also underpin anxiety vulnerability. For instance, attentional bias away from positive stimuli has been found to mediate the relationship between social anxiety and stress in response to a speech task [14]. Social anxiety symptom reduction following cognitive behavioural group therapy has been associated with an increase in attention to positive stimuli [15], and a greater readiness to acquire an attentional bias towards positive stimuli has been shown to predict lesser anxious reactivity to a subsequent stressor [16]. Taken together, the deficient attentional processing of positive stimuli may be a critical contributor to the maintenance of heightened trait anxiety.

In addition to the associations between anxiety and the biased attentional processing of threat and positive information, further research has sought to assess the mechanisms which contribute to attentional bias. Neurocognitive models posit that selective attention is controlled by two biasing signals from a bottom-up stimulus driven system and a top-down attentional control system [17]. The stimulus driven system is thought to be largely amygdala-centred [18]. Although the amygdala is well established for its role in threat detection, research suggests that the amygdala is additionally recruited for the processing of positive rewarding stimuli [19]. Hence, the amygdala may function to initially deploy attention automatically to salient emotional stimuli [20]. In conjunction, the latter attentional control system, incorporating areas such as the lateral prefrontal cortex (lPFC) and rostral anterior cingulate cortex (rACC), may then provide a more flexible goal directed control of attention relevant to task demands, including the inhibition of task irrelevant stimuli [18].

The antisaccade task [21] is a well established method for examining attention control in a range of psychopathology [22]. In this task, participants are presented with a peripheral stimulus, to which they are required to either prosaccade (look towards) or antisaccade (look away) from the stimulus. Saccade mean latency and error rates are commonly calculated as dependent measures. The prosaccade is largely a reflexive response, providing an assessment of stimulus driven attentional capture. By contrast, the correct execution of the antisaccade requires both the inhibition of the automatic prosaccade and the subsequent generation of a volitional saccade away from the distracter stimulus [23]. Critically, correct antisaccade performance necessitates prefrontal regions [22]–[24]. Hence, antisaccade performance may provide a useful assessment of inhibitory attentional control.

An emotional variant of the antisaccade task has been used [25] to further assess stimulus driven and attentional control biases towards and away from positive and threat valenced stimuli. Anxious individuals have been found to be slower to antisaccade away from threat stimuli, suggesting a specific impairment in the attentional inhibition of such stimuli [25], [26]. Clinically anxious adolescents have been found to make speeded prosaccades to threat [27]. In this study, low anxious adolescents additionally exhibited reduced antisaccade errors for threat and positive stimuli, relative to neutral stimuli, while this effect was absent in anxious adolescents [27]. A further study observed that anxious individuals made greater antisaccade errors, although this was not influenced by the valence of the stimulus [28]. Interestingly, while accumulating evidence suggests that anxiety is associated with the deficient attentional processing of positive stimuli [10]–[16], anxiety-linked differences for positive stimuli have not typically been observed in previous antisaccade studies [25], [26], [28].

In addition to latency and error rate measures, saccade peak velocity may further provide an assessment of the neurocognitive processes during antisaccade performance. Recent research suggests that saccade peak velocity may index the cognitive load experienced during a complex task, whereby decreased peak velocity is associated with greater cognitive load [29], [30]. It has been suggested that peak velocity may reflect the cognitive effort recruited to perform the antisaccade [31].

Jazbec *et al.* [32] assessed anxiety group differences on antisaccade performance, with the addition of incentive reward and punishment conditions. In particular, the peak velocity of erroneous antisaccades was used as an assessment of compensatory efforts made to attenuate the error, such that decreased peak velocity reflected greater compensatory effort. Anxious individuals were found to lack the normative decrease in peak velocity in response to incentive, compared to neutral, antisaccade conditions. While Jazbec *et al.* [32] examined antisaccade peak velocity in response to a neutral stimulus target, the addition of emotional stimuli may further be of interest. Given that anxiety may be marked by a particular difficultly in the inhibition of threat processing [25], [26], it is possible that anxiety may be associated with an attenuated peak velocity for antisaccades in response to threat. However, no study has examined anxiety group differences in antisaccade peak velocity using emotional stimuli.

Hence, the present study sought to examine the mechanisms which contribute to anxiety-linked attentional bias using an emotional antisaccade task. In light of previous research [25], it was first predicted that anxious individuals would be slower to antisaccade from threat stimuli. Given this anxiety-linked difficulty in the inhibition of threat processing, it was additionally predicted that anxiety would be associated with reduced peak velocity for erroneous antisaccades in response to threat stimuli. Finally, given previous research which has shown that anxiety has been associated with the deficient attentional processing of positive stimuli [8], [13], we sought to examine whether high anxious individuals would exhibit any selective processing biases in either pro- or antisaccade performance in relation to positive stimuli.

## Method

### Ethics Statement

All participants provided informed written consent, and were fully debriefed following the experiment. Data collected from participants were de-identified. This research was conducted in accordance with the principles expressed in the Declaration of Helsinki, and ethical approval was granted by the Human Research Ethics Committee, University of Western Sydney.

### Participants

Fifty-nine participants were initially recruited from the University of Western Sydney. Participants consisted of staff and student members of the university who responded to an internal advertisement. Three participants were removed due to significant calibration difficulties and one further participant failed to complete the task as required. The remaining fifty-five participants (39 female) were included for analysis. Participants were assigned to either low (*n* = 29) or high trait anxious (*n* = 26) groups based on a median split (median = 40) of their scores on the State Trait Anxiety Inventory Trait Version (STAI-T) [33]. All participants had correct or corrected-to-normal vision, and were reimbursed with either course credit or a $15 gift voucher.

### Measures

#### Questionnaires

Participants completed the STAI-T in order to assess trait anxiety. Reliability and validity across a broad range of populations, including undergraduate students, has previously been demonstrated [33], [34].

#### Experimental hardware

The experimental task was developed using Experiment Builder 1.10.165 (SR Research Ltd, Mississauga, Canada), and presented on a 20″ CRT monitor at a resolution of 1280×1024. Participants' eye movement was recorded using a desktop mounted SR Research EyeLink 1000. Using pupil centre corneal reflection, the EyeLink 1000 recorded monocular gaze at 1000 Hz, with up to .25° accuracy and .01° spatial resolution. Nine calibration points were used.

#### Task stimuli

The emotional stimuli used consisted of six male and six female actors, each expressing happy, angry and neutral emotions. Faces were drawn from the NimStim Set of Facial Expressions [35]. High reliability and validity for the categorization of these facial expressions have been demonstrated [35]. Face images were presented in grayscale and were 6.13 cm by 7.87 cm (width by height) subtending at approximately 6.15° by 7.90° visual angle (VA) respectively. Face images were additionally mean luminance and contrast matched using the Spectrum Histogram and Intensity Normalization and Equalization toolbox (SHINE) [36].

#### Antisaccade task

Each trial commenced with the presentation of a fixation cross for 1500 ms. The fixation cross was white, positioned at the display centre, and subtended at approximately 1° VA, at a viewing distance of 57 cm. This was replaced by a positive, neutral or threat face, presented at 11° VA eccentricity to the left or right of display centre. Progression of the trial was contingent upon participants' gaze such that at the end of the 1500 ms fixation cross presentation, the target face would only appear if gaze was located at the cross. Otherwise, the fixation cross would remain on the screen until gaze was detected. The target face then remained on screen for 600 ms before being extinguished. Participants were required to perform the appropriate saccade within this interval. Following a 500 ms inter-trial interval, the next trial was presented.

**Figure 1:**
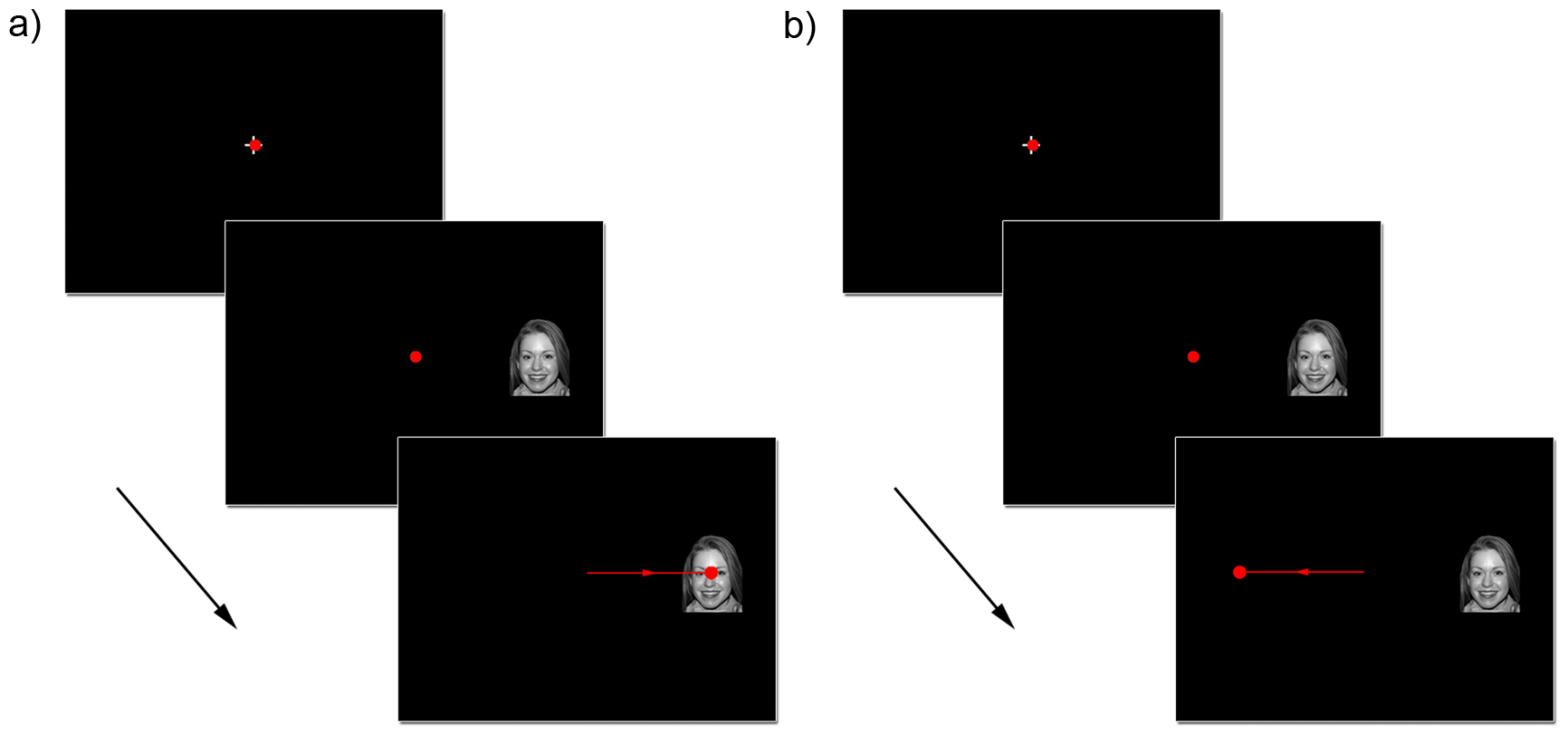
A. Trial structure. For a prosaccade trial, gaze is initially directed to a central fixation cross. Following the subsequent onset of the peripheral stimulus, a saccade is made towards this stimulus. **b**. For an antisaccade trial, gaze is similarly secured at the initial fixation cross. Following the onset of the peripheral stimulus, a saccade is made away from the stimulus. The face stimulus depicted is from the NimStim Face Stimulus Set. Reprinted with permission (http://www.macbrain.org/resources.htm).

A total of 144 trials were presented over six blocks, with three requiring antisaccades and three requiring prosaccades. Blocks were randomized with a maximum run length of two. At the start of each block, the instruction “TOWARDS” or “AWAY” was presented, indicating the type of saccade that was required for the block. Twenty-four randomized trials were presented each block, containing eight positive, eight neutral and eight threat trials. Gender and left-right location of the face stimulus were balanced. Pro- and antisaccade trials are illustrated in Figure 1.

### Procedure

Participants were initially informed that the present study examined attentional processes using eye tracking. Upon obtaining written consent, participants completed the STAI-T and demographic information. Participants were then seated in front of the EyeLink 1000 and their head positioned in a chin rest. Participants were informed that they would be presented with a number of trials in which they were required to either look towards or away from a peripherally appearing face. If the instruction was “TOWARDS,” participants were required to simply look at the face. If the instruction was “AWAY,” participants were required to look to the opposite side of the screen, aiming for the approximate mirror position, without looking at the face. Speed and accuracy were emphasized. After participant comprehension of the task was confirmed by the experimenter, participants were calibrated and then completed 12 practice trials. At the end of each practice trial, “Correct” or “Incorrect” was presented for 1 s in green or red respectively, providing per-trial feedback. Participants then completed the 144 experimental trials. The task was completed in a dimly lit sound attenuated room. Participants' eye movements were monitored from a second room, and recalibrations were performed if required. Participants were subsequently debriefed and reimbursed.

### Data Preparation

Saccades were defined as eye movements above a 30°s^−1^ (degrees per second) velocity threshold and 8000°s^−2^ (degrees per second squared) acceleration threshold. In order to remove anticipatory saccades and artifactual gaze data, trials were included for analysis if the first saccade following target onset was (a) greater than 3° amplitude, (b) occurred between 83–600 ms following target onset, and (c) directed within 45° from horizontal. To assess task performance, mean latency and error rates were calculated for pro- and antisaccades from positive, neutral and threat stimuli. Latency to perform the instructed saccade was calculated from correct response trials, and indexed as the time between face onset and the initiation of the correct saccade. Pro- and antisaccade error rates were defined as the proportion of trials with incorrect saccade responses relative to the total usable trials otherwise satisfying the aforementioned trial inclusion criteria. The mean peak velocity of correct pro- and antisaccades, and erroneous antisaccades was further calculated for positive, neutral and threat stimuli. The peak velocity of erroneous prosaccades was not considered as this rarely occurred (see prosaccade error rates in Table 1).

**Table 1:** Means and standard deviations of pro- and antisaccade latency and error rates for high and low anxious participants.

Variable		High		Low	
Type	Valence	*M*	*SD*	*M*	*SD*
Prosaccade					
Latency (ms)	Positive	126.82	17.61	136.04	25.68
	Neutral	127.54	18.11	133.08	20.73
	Threat	127.14	19.42	133.50	22.68
Error Rate (%)	Positive	0.00	0.00	0.00	0.00
	Neutral	0.00	0.00	0.00	0.00
	Threat	0.00	0.00	0.20	1.09
Antisaccade					
Latency (ms)	Positive	210.92	35.25	223.55	40.70
	Neutral	216.19	38.24	218.44	37.28
	Threat	214.42	38.15	216.95	40.81
Error Rate (%)	Positive	11.01	8.12	12.10	13.54
	Neutral	10.02	12.04	12.79	12.67
	Threat	11.03	10.48	13.71	14.02

## Results

### Group Characteristics

A one way ANOVA confirmed that high anxious participants reported higher STAI-T scores, *F*(1,53) = 107.56, *p*<.001, in comparison to low anxious participants. In addition, no group differences in mean age (*M* = 21.08 years, *SD* = 3.05), *F*(1,53) = 1.21, *p* = .276, or gender, χ^2^(1, *N* = 55) = .87, *p* = .389, were evident.

### Saccade Latency

**Figure 2:**
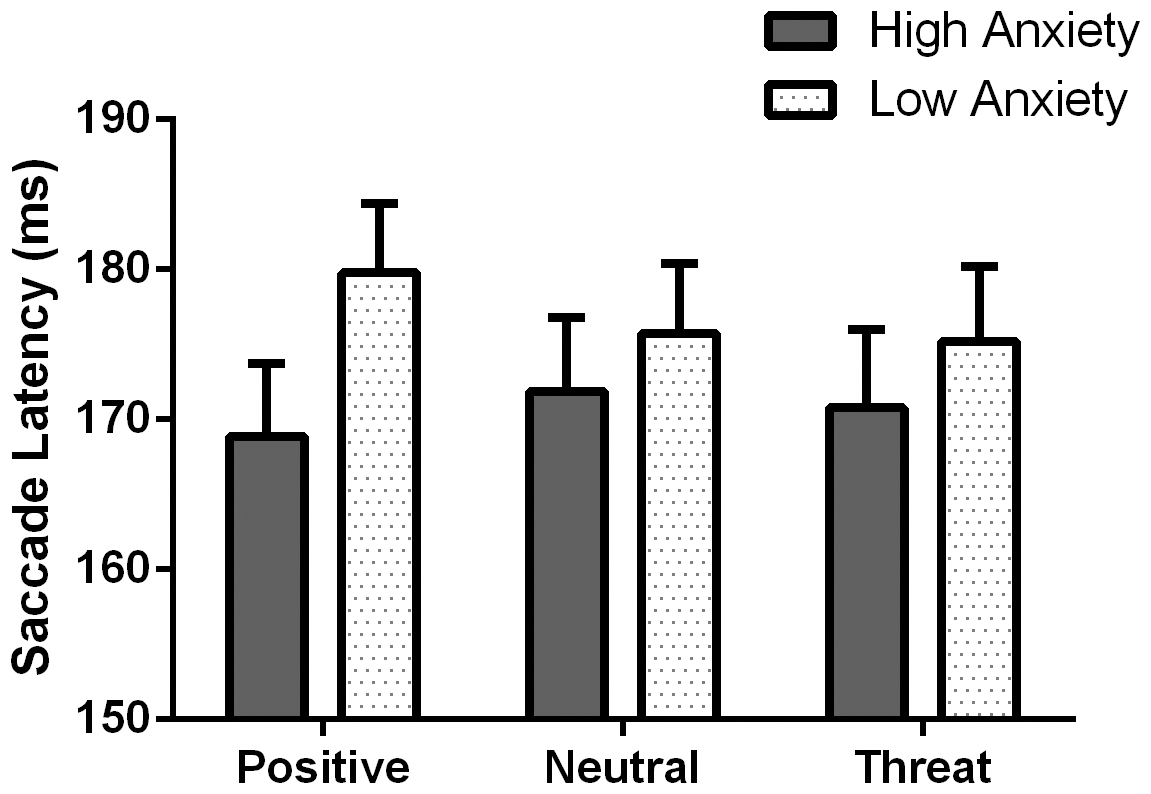
Saccade latency data. Mean saccade latencies for positive, neutral and threat stimuli for high and low anxious participants. Error bars represent the standard error.

To assess differences in mean saccade latency of correct responses, a group (high vs. low) by saccade (pro- vs. antisaccade) by valence (positive vs. neutral vs. threat) mixed design ANOVA was conducted. A main effect of saccade was evident, *F*(1,53) = 363.44, *p*<.001, partial η^2^ = .87, indicating that prosaccades were faster compared to antisaccades (*M* = 130.69, *SD* = 20.27). Importantly, a group by valence interaction was significant, *F*(2,106) = 4.27, *p* = .016, partial η^2^ = .08, illustrated in Figure 2. Pairwise comparisons using Bonferroni adjustments were used to further clarify this interaction. Within-subjects comparisons confirmed that low anxious participants were slower to saccade either toward or away from positive stimuli compared to threat, *M*_diff_ = 4.57, *SE*_diff_ = 1.60, *p* = .018. However, high anxious participants showed no difference, largest *M*_diff_ = −3.00, *SE*_diff_ = 1.93, *p* = .377. No other within-subject effects were significant, largest *F*(2,106) = 1.14, *p* = .324. No between-subject Bonferroni adjusted pairwise comparisons were significant, largest *M*_diff_ = 10.92, *SE*_diff_ = 6.69, *p* = .109.

### Saccade Error Rates

A group (high vs. low) by saccade (pro- vs. antisaccade) by valence (positive vs. neutral vs. threat) mixed design ANOVA was conducted on saccade error rates. A main effect of saccade was found, *F*(1,53) = 67.02, *p*<.001, partial η^2^ = .56, indicating that participants had a greater proportion of errors for antisaccades (*M* = 11.78, *SD* = 10.81) compared to prosaccades (*M* = .03, *SD* = .27). No other effects were observed, largest *F*(1,53) = .58, *p* = .452.

### Peak Velocity of Correct Saccades

To assess for peak velocity differences for correct pro- and antisaccades, a group (high vs. low) by saccade (pro- vs. antisaccade) by valence (positive vs. neutral vs. threat) mixed design ANOVA was conducted. Mauchly's test indicated a violation of the assumption of sphericity, *χ^2^*(2) = 36.19, *p*<.001, hence Greenhouse-Geisser adjusted values have been reported (*ε* = .67). A main effect of saccade was observed, *F*(1,53) = 25.95, *p*<.001, partial η^2^ = .33, indicating that prosaccades (*M* = 431.13, *SD* = 68.84) had a higher peak velocity compared to antisaccades (*M* = 398.10, *SD* = 79.34). A saccade by valence interaction emerged, *F*(1.33,70.60) = 3.78, *p* = .044, partial η^2^ = .07. While the largest mean difference, derived from pairwise comparisons using Bonferroni corrections, suggested that antisaccade peak velocity may have been faster for threat (*M* = 404.49, *SD* = 88.61) compared to neutral (*M* = 395.19, *SD* = 77.08) stimuli, *M*_diff_ = 9.30, *SE*_diff_ = 4.41, *p* = .119, no pairwise comparisons reached statistical significance. A valence trend was observed, *F*(1.58,83.85) = 2.59, *p* = .093, partial η^2^ = .05. However, pairwise comparisons using Bonferroni corrections revealed no significant differences, largest *M*_diff_ = 4.32, *SE*_diff_ = 2.37, *p* = .223. No other effects were evident, largest *F*(1,53) = 2.70, *p* = .106.

### Peak Velocity of Erroneous Antisaccades

**Figure 3:**
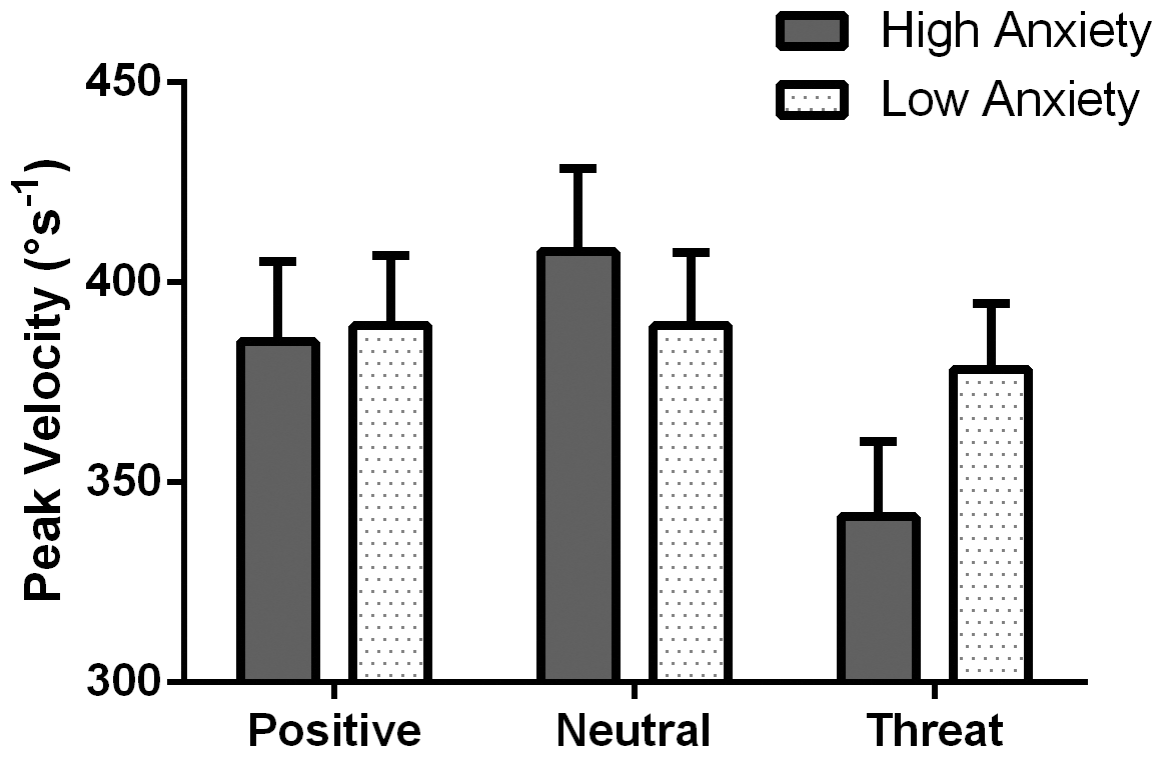
Peak velocity data. Mean peak velocities for erroneous antisaccades in response to positive, neutral and threat stimuli for high and low anxious participants. Error bars represent the standard error.

To examine for compensatory efforts in response to erroneous antisaccades, a group (high vs. low) by valence (positive vs. neutral vs. threat) mixed design ANOVA was run on peak velocity. Some participants did not make erroneous antisaccades in response to all positive, neutral and threat stimuli, and were therefore not able to be included. The analysis was conducted on the remaining 17 low and 13 high anxious participants. A main effect of valence was found, *F*(2,56) = 4.93, *p* = .011, partial η^2^ = .15. Pairwise comparisons using Bonferroni adjustments confirmed that participants showed slower peak velocities for threat compared neutral stimuli, *M*_diff_ = 38.56, *SE*_diff_ = 14.87, *p* = .045. Interestingly, a possible group by valence interaction emerged at trend level, *F*(2,56) = 2.43, *p* = .098, partial η^2^ = .08. Exploratory follow-up pairwise comparisons were conducted using Bonferroni adjustments. High anxious participants were found to show significantly reduced peak velocity for threat compared to neutral stimuli, *M*_diff_ = 66.21, *SE*_diff_ = 22.39, *p* = .019. However, no differences were evident in low anxious participants, largest *M*_diff_ = 10.92, *SE*_diff_ = 19.58, *p* = 1.000. Figure 3 illustrates this interaction trend. Peak velocity descriptive statistics are detailed in Table 2.

**Table 2:** Peak velocity means and standard deviations for correct prosaccades, and correct and erroneous antisaccades for high and low anxious participants.

Variable		High		Low	
Type	Valence	*M*	*SD*	*M*	*SD*
Prosaccade					
Correct	Positive	431.93	60.14	430.11	75.92
	Neutral	433.65	60.88	431.55	74.65
	Threat	429.25	62.12	430.31	76.15
Antisaccade					
Correct	Positive	384.22	68.13	405.00	85.00
	Neutral	383.83	64.37	406.54	86.68
	Threat	395.67	79.06	413.31	96.10
Erroneous	Positive	385.15	50.06	389.15	86.17
	Neutral	407.63	77.93	389.18	73.67
	Threat	341.42	75.26	378.27	61.22

Peak velocity values are given in degrees per second.

## Discussion

The present study sought to elucidate the mechanisms of biased selective attention to emotional stimuli in anxiety, by implementing an antisaccade task. Saccade performance was found to be influenced by trait anxiety. While low anxious individuals were found to be slower to perform saccades in response to positive stimuli, irrespective of whether pro- or antisaccades were required, high anxious individuals, by comparison, were relatively faster to perform such saccades. Such a difference may suggest that trait anxiety may influence the way in which positive stimuli is initially processed before the execution of either the pro- or antisaccade. The findings add to the nascent literature suggesting that high trait anxiety is associated with the deficient attentional processing of positive information [7], [8].

It has been suggested that information which is preferentially attended to may be congruent to an individual's emotional disposition or state [9]. Consistent with this, low anxious individuals have been found to preferentially attend to positive information [37], [38], while high trait anxiety has been associated with a reduced attentional preference for positive stimuli [10]–[13]. It is possible that reduced processing may similarly occur for anxious individuals during the early processing of positive information before a saccade response is made. For instance, when a positive stimulus is peripherally presented, covert attentional processing is initially required before the execution of both pro- and antisaccades. It is possible that deficient processing of positive stimuli during this early stage may subsequently shorten the latency before the pro- or antisaccade is initiated, as observed in the present study.

Previous research suggests that anxiety may be associated with the aberrant processing of positive information. For instance, socially anxious individuals have been shown to appraise happy faces as being less approachable [39], and have exhibited reduced behavioural approach to positive stimuli [40]. It has been suggested that social anxiety may be characterized by a fear of positive social evaluation, in which positive social evaluation is feared as it is perceived to lead to undeservedly high regard and subsequent conflict with others [41]. Similarly it has been suggested that socially anxious individuals may perceive positive social events as threatening, as such events may be taken to convey heightened and unachievable social expectations which will subsequently lead to social failure [42]. It is possible that such dysfunctional perceptions of positive information may stem from aberrations in the early processing, such as the present finding of anxiety-linked reduced saccade latency in response to positive stimuli.

The present study additionally sought to examine anxiety-linked differences in the peak velocity of saccades performed in response to emotional stimuli. Previous research has shown that peak velocity may be sensitive to cognitive processes, such that a relative reduction in peak velocity may reflect an increased cognitive effort recruited to perform the antisaccade [29]–[31]. Of particular interest, the peak velocity of erroneous antisaccades was analyzed to assess for compensatory efforts made in response to error. While the overall analysis was not significant at the .05 level, exploratory follow up comparisons suggest that high anxious individuals may have shown a reduction in peak velocity when incorrectly saccading towards threat, relative to neutral stimuli, while low anxious individuals did not show this dependence on valence. This reduction in peak velocity has been suggested to reflect an attempt to attenuate the erroneous saccade after the movement is initiated [32]. The finding tentatively suggests that anxious individuals may have exerted greater compensatory efforts in response to failing to appropriately inhibit the threat stimulus. Such a notion is consistent with previous literature which suggests that anxiety is marked by a particularly difficulty in the inhibition of threat processing [43], [44].

Peak velocity of correct saccades was additionally examined. Antisaccade peak velocity was found to be reduced compared to prosaccades as expected [45]. Differences between peak velocities in response to positive, neutral and threat stimuli may have varied between pro- and antisaccades for all participants. However, no significant pairwise differences were evident, precluding any strong interpretations. No significant influence of anxiety was evident. Hence, the present findings suggest that peak velocity may be a useful measure of the compensatory processes of erroneous antisaccades. The possible finding that threat valence may modulate peak velocity in anxiety, extends previous research which only examined erroneous antisaccade peak velocity for neutral stimuli [32]. However, further replication is required.

While trend level anxiety group differences were found for erroneous antisaccades in response to threat, anxiety was not found to influence correct antisaccades from threat. This was somewhat unexpected given previous findings associating anxiety with slower latencies for correct antisaccade in response to threat [25], [26]. However, it is to be noted that other previous research similarly failed to replicate a threat specific bias in antisaccade performance for anxious individuals [28]. While it has been posited that anxiety may be marked by an impairment in the inhibition of threat distracters [43], research further suggests that anxious individuals may recruit greater cognitive resources in order to compensate for this impairment [46], [47]. It is plausible that an increase in compensatory effort in anxious individuals may have ameliorated any anxiety group differences in antisaccade latency in response to threat. Moreover, such increased compensatory effort may have been evidenced by the reduced peak velocity to threat observed in anxious individuals.

The findings of the present study suggest that threat may have influenced erroneous but not correct antisaccades. Given this moderating influence of the correctness of the antisaccade execution, it is possible that task difficulty may account for these findings. Working memory capacity, as assessed by the operation-span task (OSPAN), has previously been shown to influence antisaccade performance [48]. Similarly, a recent study by Berggren *et al.* [49] investigated the effect of cognitive load and anxiety on antisaccade performance. Increased cognitive load was associated with longer antisaccade latencies, and this was particularly marked for high anxious individuals. Stimulus valence was not found to influence this anxiety-linked effect. However, peak velocity was not examined.

Future research may benefit from examining the task designs optimal for elucidating the biases associated with anxiety, while including the assessment of erroneous antisaccade peak velocity. For instance, increasing the task difficulty, by including a secondary task [49], by incorporating a greater number of stimulus locations, or a brief, yet variable, gap between the offset of the fixation cross and the target onset, may result in greater discrimination of antisaccade performance. Moreover, a limitation of the present study was that not all participants made erroneous antisaccades, which restricted the analysis of erroneous antisaccade peak velocity, and the strength of conclusions which may be drawn. A comparatively difficult antisaccade task would result in more instances of erroneous antisaccades, which in turn, would enable a more powerful analysis.

The present study sought to examine the mechanisms of attentional bias to emotional stimuli in anxiety. Low anxious individuals were found to be slower to saccade in response to positive stimuli, irrespective of whether a pro- or antisaccade was required. However, such a positivity bias was absent for high anxious individuals. Anxiety may have additionally been associated with the reduced peak velocity of erroneous antisaccades in response to threat, suggesting that anxious individuals may have exerted greater compensatory efforts in the inhibition of threat. The findings highlight that alternate saccadic measures, such as peak velocity, may provide useful insights into the biased attentional processes which characterize anxiety.
